# Convergence of therapy-induced senescence (TIS) and EMT in multistep carcinogenesis: current opinions and emerging perspectives

**DOI:** 10.1038/s41420-020-0286-z

**Published:** 2020-06-15

**Authors:** Mir Mohd Faheem, Nathan D. Seligson, Syed Mudabir Ahmad, Reyaz Ur Rasool, Sumit G. Gandhi, Madhulika Bhagat, Anindya Goswami

**Affiliations:** 1 Cancer Pharmacology Division, CSIR-Indian Institute of Integrative Medicine, Jammu, 180001 India; 2 School of Biotechnology, University of Jammu, Jammu, 180006 India; 3 Department of Pharmacotherapy and Translational Research, The University of Florida, Jacksonville, FL USA; 4 Department of Pharmacogenomics and Translational Research, Nemours Children’s Specialty Care, Jacksonville, FL USA; 5 Academy of Scientific & Innovative Research (AcSIR), CSIR- Indian Institute of Integrative Medicine, Jammu, 180001 India; 6 Perelman School of Medicine, Cancer Biology Division, University of Pennsylvania, Philadelphia, PA 19104 USA; 7 Plant Biotechnology Division, CSIR-Indian Institute of Integrative Medicine, Jammu, 180001 India

**Keywords:** Metastasis, Cancer therapy

## Abstract

Drug induced resistance is a widespread problem in the clinical management of cancer. Cancer cells, when exposed to cytotoxic drugs, can reprogram their cellular machinery and resist cell death. Evasion of cell death mechanisms, such as apoptosis and necroptosis, are part of a transcriptional reprogramming that cancer cells utilize to mediate cytotoxic threats. An additional strategy adopted by cancer cells to resist cell death is to initiate the epithelial to mesenchymal transition (EMT) program. EMT is a trans-differentiation process which facilitates a motile phenotype in cancer cells which can be induced when cells are challenged by specific classes of cytotoxic drugs. Induction of EMT in malignant cells also results in drug resistance. In this setting, therapy-induced senescence (TIS), an enduring “proliferative arrest”, serves as an alternate approach against cancer because cancer cells remain susceptible to induced senescence. The molecular processes of senescence have proved challenging to understand. Senescence has previously been described solely as a tumor-suppressive mechanism; however, recent evidences suggest that senescence-associated secretory phenotype (SASP) can contribute to tumor progression. SASP has also been identified to contribute to EMT induction. Even though the causes of senescence and EMT induction can be wholly different from each other, a functional link between EMT and senescence is still obscure. In this review, we summarize the evidence of potential cross-talk between EMT and senescence while highlighting some of the most commonly identified molecular players. This review will shed light on these two intertwined and highly conserved cellular process, while providing background of the therapeutic implications of these processes.

## Facts

- Multiple signaling pathways are shared between TIS and EMT.
- Metastatic re-programing initialized through epithelial to mesenchymal transition (EMT) represents an aggressive process associated with significant mortality in cancer.
- Therapy-induced senescence (TIS) provides a practical approach to cancer management with improved patient prognosis in clinical settings.

## Open questions

- Is activation of EMT and inhibition of senescence mutually inclusive or molecularly unlinked?
- How does contextual regulation of effectors of senescence and EMT associated transcription factors determine cellular fate?
- Can identification of novel signaling links between EMT and senescence provide rational drug targets for cancer therapy?

## Introduction

Traditional chemotherapeutic approaches have resulted in limited success in the war against cancer. Cytotoxic drugs inflict harm on both cancer cells as well as normal cells; therefore, the complete eradication of cancer cells by cytotoxic drugs within a solid tumor may not be realistically possible without causing significant adverse side effects in patients. Recent evidence suggests that such treatments may trigger resistance in cancer cells; leading to more frequent relapse and progression to metastatic disease^1–3^. Alternatively, targeting the proliferative capacity of cancer cells to trap them in a permanently growth arrested or cytostatic state, without stimulating cell death pathways, has shown promising results in preliminary clinical investigations^4^. Cellular senescence, a type of cytostasis, is characterized by irreversible growth arrest with distinct molecular and morphological phenotypes^5,6^. The seminal finding by Hayflick et al. that in vitro cell cultures lose their replicative capacity over time has led to the development of the field of senescence^7^. This was later attributed to cellular aging due to telomere attrition.

Although senescence is primarily the cause of cellular aging, it occurs transiently during embryogenesis and organism development as well as during tissue remodeling processes such as wound healing. In these cases, senescence serves as a mechanism to identify and prepare cells which are no longer biologically necessary for rapid clearance by the immune system. Senescence can be triggered prematurely by a variety of stress signals, including hyper-activated oncogenic signaling. This process, known as oncogene-induced senescence (OIS), relies on the contextual pre-activation of certain oncogenes and is compensated by downstream activation of tumor-suppressors of retinoblastoma protein (Rb) and p53; culminating in cell cycle arrest^8^. Loss of critical tumor suppressors, such as PTEN, can also trigger premature senescence via a process termed tumor suppressor loss-induced senescence (TSLIS)^9^. In cancer cells, premature senescence can be induced by certain therapeutic agents and is referred to as therapy-induced senescence (TIS). Targeting TIS as a therapeutic goal represents a functional strategy in cancer therapeutics that may improve patient prognosis by conferring the added advantage of a reduced side effect profile compared with cytotoxic agents^4^. This approach is novel in that while it may not promote the eradication of a cancer, it provides a pragmatic goal with regards to disease management and patient survival akin to chronic disease management^10^.

Cancer cells frequently alter their ‘omic’ landscape and exhibit profound changes in their secretome^11^. Successively, vital cytokines (IL-6, IL-8, TNF-α, TGF-β, etc.) and chemokines (CXCR2, etc.) are released into the extracellular milieu. Notably, cells with strong activation of DNA damage responses (DDR) turn on accelerated senescence signaling leading to fundamental changes in secretome^12^, referred to as senescence-associated secretory phenotype (SASP). Persistent DNA damage is pre-requisite for activation of SASP factors. As DNA damage precedes some but not all types of senescence, SASP is not observed in all senescent settings^13^. SASP can be beneficial to the host organism over a short period of time in a cell-autonomous manner by limiting proliferation and fibrosis. However, long-term activation of SASP activates a plethora of non-cell-autonomous cross-talk, which can have pro-oncogenic effects in pre-tumorigenic cells and fibroblasts within the tumor microenvironment (TME). These effects include chronic inflammation and initiation of epithelial to mesenchymal transition (EMT)^14^. EMT, just like senescence, is an evolutionarily conserved process conferring a vital role in maintaining tissue homeostasis and remodeling. However, the evidence for the involvement of EMT in various pathogenic processes is mounting^15^. The initiation of EMT, due to exposure to anti-cancer therapeutic agents, causes cancer cells to be more invasive and prone to metastasis. While the association of cellular senescence with EMT is still developing, an in-depth understanding of proposed shared mechanism for these inter-twined physiological processes is warranted.

This review serves to provide a comprehensive overview of recent developments in the field of TIS, associated molecular mechanisms, and the potential of senescence as a therapeutic goal for cancer. Special emphasis is also provided regarding the convergence of TIS and EMT; which are highly involved in carcinogenesis and are now believed to be inextricably linked.

## Mechanisms involved in TIS

**Fig. 1:**
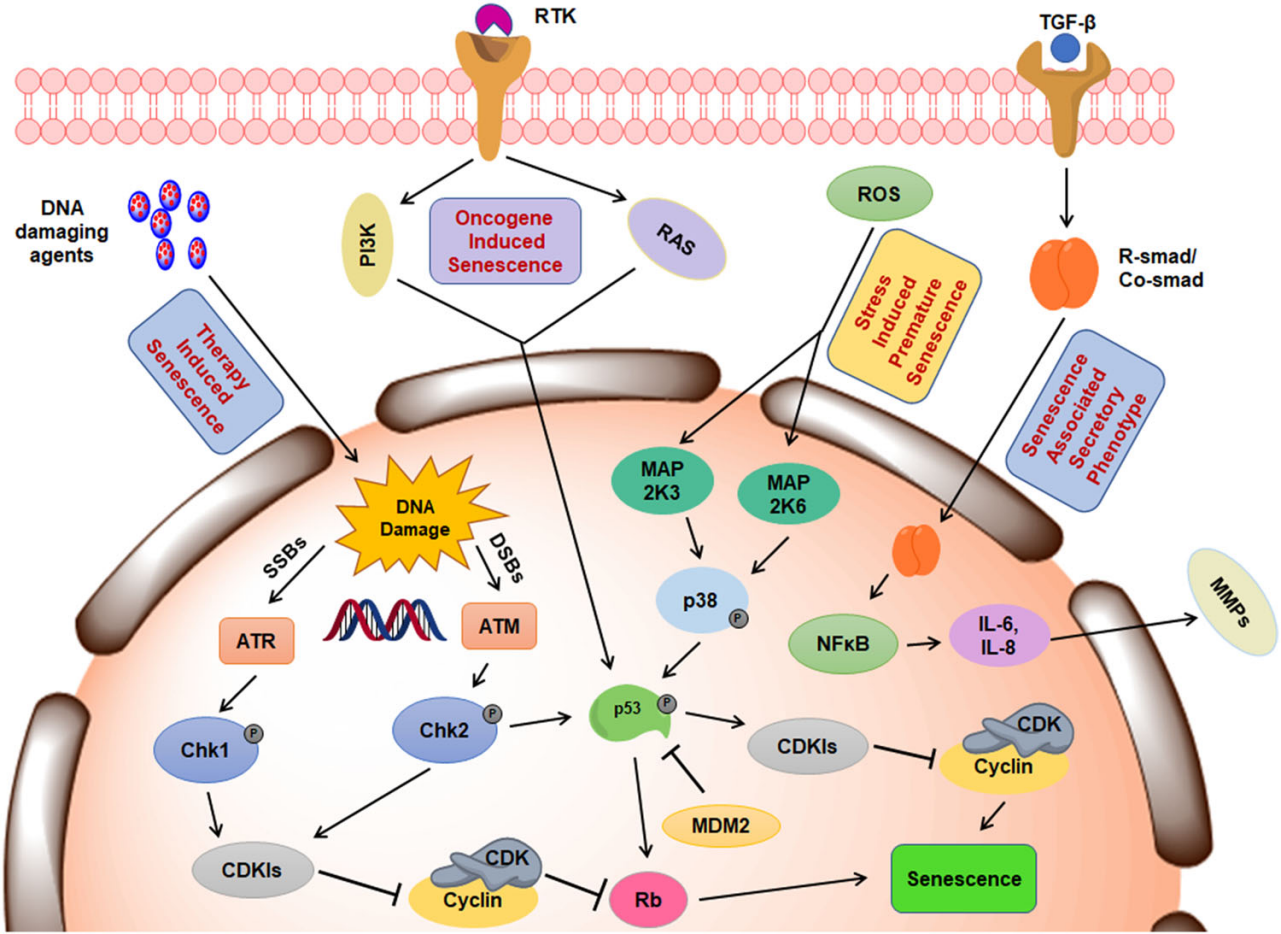
Schematic elucidating the mechanism of therapy-induced senescence. Activation of DNA damage response (DDR) pathways in response to therapy results in ATM/ATR kinase mediated induction of specific Cyclin dependent kinase inhibitors (CDKIs) that hinder the complex formation between cyclins and CDKs. Apart from DDRs, multiple signaling pathways (PI3K/AKT, Ras/MAPK, ROS/p38) are activated that converge on cell cycle checkpoints and the tumor suppressor Rb to induce senescence. Of note, certain agents can also activate the TGF-β signaling resulting in activation of cytokines (IL6, IL8) and Matrix metalloproteases (MMPs) culminating in senescence-associated secretory phenotype (SASP).

Apoptosis and senescence are partly linked by the stresses that act to induce their activation. Ultimately, the cellular decision between senescence and apoptosis is reliant on the nature, magnitude, and duration of the stress. As an example, sub-toxic exposure of cytotoxic damage rarely lead to apoptosis; rather, sub-toxic levels of cytotoxic damage results in an anti-proliferative, senescence response. Despite the loss of the capacity to divide in senescent cells, molecular changes made during senesce allows cells to persist indefinitely with somewhat compromised viability. Therefore, the selection of drugs and their dosing pose real challenges in therapeutic intervention. While apoptosis is a rapid process that can be triggered within a very short span of time (within 24 h), senescence and SASP take several days to fully activate^4^. The primary signaling mechanisms provoking TIS (Fig. 1) and their consequent outcomes in therapeutic development are summarized in the following sections.

**Fig. 2:**
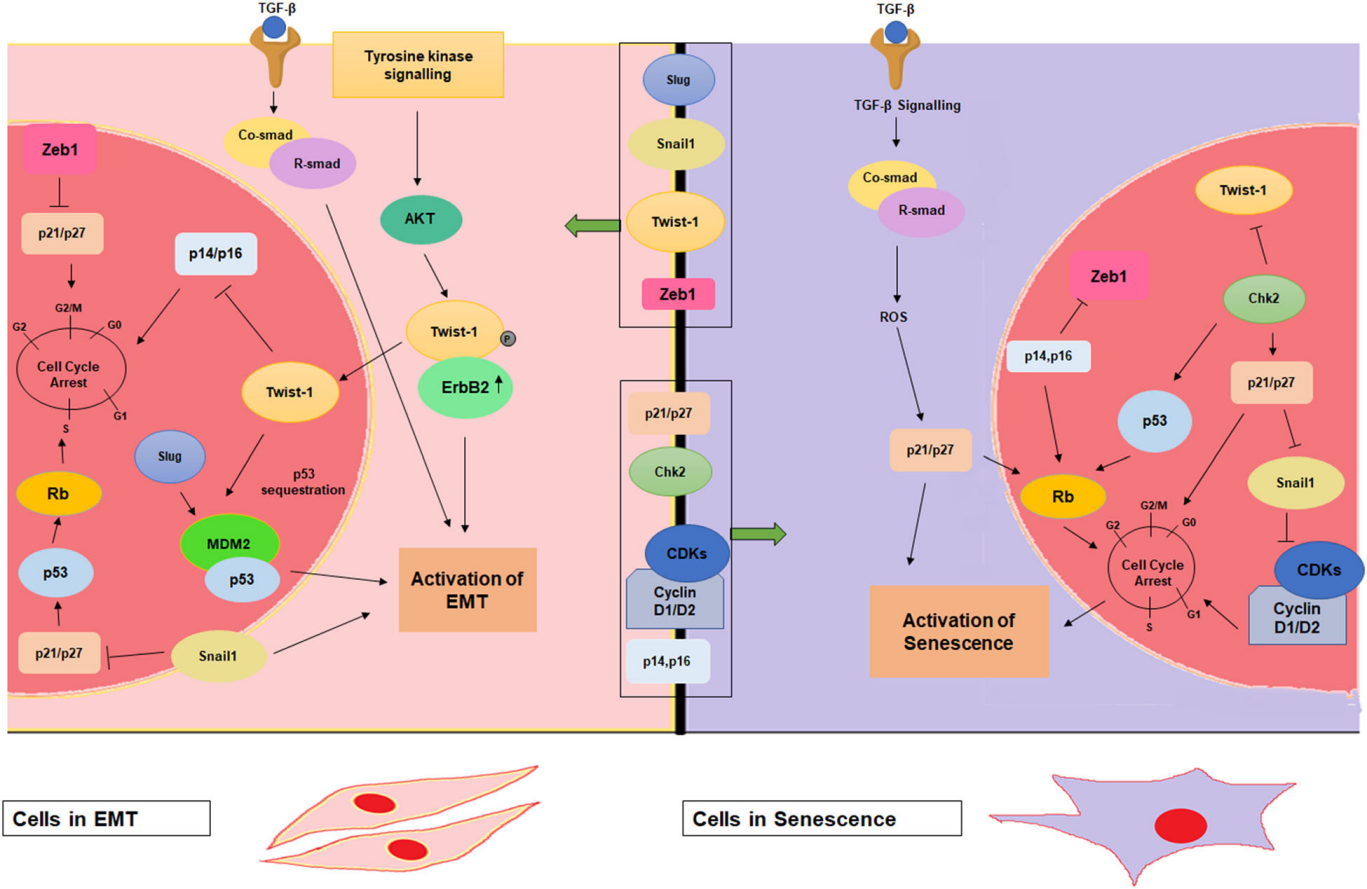
Signaling cross talks in senescence and EMT. Cells undergoing premature senescence have elevated ROS levels resulting in the induction of p21 and other cell cycle check point kinases. Various CDKIs repress the EMT-associated transcription factors like Twist-1, Zeb1 and Snail1. On the contrary, cells in EMT have diminished p21/p27 and p14/p16 levels due to activated Twist-1 levels and other EMT-associated transcription factors.

### DNA damage mediated activation

Unlike replicative senescence, which is predominantly an aging-related phenomenon characterized by telomere shortening, TIS is considered to be an accelerated form of cellular senescence in response to genotoxic stresses. In fact, majority of the TIS inducing agents are either targeted towards inflicting direct DNA damage, or directly altering the DNA landscape in terms of its structure and function, underscoring the culpability of genomic stress in the activation of senescence^16^. For example, both doxorubicin and vorinostat are potential TIS inducing agents that elicit DDR. Doxorubicin provokes double-stranded DNA breaks (DSBs) by poisoning topoisomerase II and directly damaging DNA whereas vorinostat, a pan-histone deacetylase (HDAC) inhibitor, alters the normal chromatin structure. Of note, HDACs participate in DNA repair and their depletion by RNAi or specific inhibitors leads to increased ϒH2AX expression and heightened DDR^17,18^. Consequently, DDR is orchestrated via Ataxia Telangiectasia–Mutated (ATM) and Ataxia Telangiectasia and Rad3-related (ATR) kinases. ATM and ATR serve as sensors for the detection of DSBs and single-stranded breaks (SSBs) in the DNA, respectively^19^. Following DNA damage, downstream targets of ATM and ATR are chiefly the cell cycle regulatory proteins, checkpoint homologs 1 and 2 (Chk1 and Chk2), which in turn activate various biologic cyclin-dependent kinase inhibitors (CDKIs)^20^. As a consequence, elevated expression of phase-specific CDKIs confers the arresting of cells either in the G_1_ or G_2_/M stage of cell cycle^21^. Table 1 lists the various CDKIs that serve as the effectors of senescence. Overall, the primary mediators in TIS remain the same as in other forms of senescence viz., p53, proteins encoded by the *Ink4* locus (p14, p15, p16, p18, and p19) and the Cip/Kip family of proteins (p21, p27, and p57); with p53, p16, and p21 contributing a central role in activating senescence^22^. However, the absence of these proteins does not mean that TIS cannot be activated in response to genotoxic stresses. Litwiniec et al. demonstrated that activation of TIS by etoposide in A549 cells promotes intense SA-β-gal activity; however, neither induction of p21, formation of senescence-associated heterochromatin foci (SAHF), nor a stable cell cycle arrest was identified solely owing to the homozygous loss of the *Ink4* locus in these cells^23^. Importantly, the relevance of the *Ink4* locus in inducing senescence can be attributed to the fact that its deletion predisposes the cells to tumorigenesis^24^. Taken together, the role of DDR in activating TIS is well established; however, the data are also clear that this is not the sole mechanism for achieving TIS as loss of key DDR pathways does not preclude cells from achieving TIS.

**Table 1:** Effectors of senescence.

S. No.	Effectors	Encoding gene	Interacting partners	Cyclins	Cell cycle arrest	Type of senescence	References
1	p14ARF (Ink4a)	CDKN2A	CDK4, CDK6	D	G_1_	OIS, TIS	^24,35,79^
2	p15 (Ink4b)	CDKN2B	CDK4	D	G_1_	RS, OIS	^33,80^
3	p16 (Ink4a)	CDKN2A	CDK4,CDK6	D	G_1_	OIS, RS, TIS	^32,34,81^
4	p18 (Ink4c)	CDKN2C	CDK4, CDK6	D	G_1_	RS, OIS	^82^
5	p19 (Ink4d)	CDKN2D	CDK4, CDK6	D	G_1_	OIS, TIS	^16,35^
6	p21 (Waf1, Cip1)	CDKN1A	CDK1, CDK2, CDK 4, CDK6	A, D, E	G_1_, G_1_/S	OIS, RS, SIPS, TIS	^22,69,83^
7	p27 (Kip1)	CDKN1B	CDK2, CDK4	D, E	G_1_, G_1_/S	OIS, SIPS, TIS	^84,85^
8	p57 (Kip2)	CDKN1C	CDK2, CDK3	E	G_1_, G_1_/S	SIPS, TIS	^86,87^

*CDK* cyclin dependent kinase, *OIS* oncogene-induced senescence, *RS* replicative senescence, *SIPS* stress induced premature senescence, *TIS* therapy-induced senescence.

### Cell cycle mediated activation

Additional regulation of TIS can be mediated by the Rb protein. Rb is a cell cycle regulatory protein which in its hypo-phosphorylated form is bound to the E2F family of transcription factors and restricts the entry of cells into S phase^25^. Induction of p16 and concomitant hyperactivation of p53 by TIS agents retains Rb in its hypo-phosphorylated form; culminating in senescent phenotype^26,27^. As mentioned above, TIS can incite DDR pathways while downstream ATM phosphorylates p53 at ser15 residue to activate senescence^28^. Phosphorylation at ser15 stabilizes p53 and prevents its sequestration by MDM2 resulting in its transactivation and elevated expression of CDKIs^29,30^. Further, p53, along with p16 and Rb, act as a base for activation of additional pro-senescent signals^31^. Rather than acting in isolation, significant cross talk is mediated between p53 and Rb to achieve senescence activation in response to chemotherapy.

Apart from activating DDR, TIS agents can stimulate reactive oxygen species (ROS) generation, unscheduled oncogene activation, and telomere dysfunction; all of which can be thought of as subsidiary mechanisms of senescence initiation^4^. Intriguingly, p16 driven hypo-phosphorylation of Rb serves as a terminal signal. It has been demonstrated that a strict correlation between elevated p16 expression and persistent cell cycle arrest continues through induction of p16 in response to extracellular stress signals mediated by p38-MAPK pathway^32^. Similarly, p21 and p15 induction, either directly or via the p53 route in response to therapy, has been identified to cause senescence activation^33^. While p53 and p21 are mainly associated with the initiation of the senescent program, p16 instead contributes to maintaining the senescent phenotype^34^. Additionally, p14^ARF^ and its murine counterpart, p19^ARF^, are primarily responsible for the sequestration of MDM2, the principal cellular regulator of p53^35^. MDM2 antagonists (Nutlin-3a and MI-63) have been illustrated to increase p53 activity leading to the abrogation of SASP^36^. Interruption of MDM2 stabilization by HDAC2 re-activates p53 signaling; demonstrating the complex network involved in its regulation^37^. Of note, mutant p53 has been positively correlated with Twist-1 expression, which serves a master regulator of EMT-associated genes and acts as a transcriptional repressor of ARF^38,39^. Attenuation of Twist-1 by Chk2 induction incites premature senescence in p53 defective cancer cells^40^.

The data here clearly suggests that multiple interconnected molecular networks are actively involved in TIS signaling. Despite these complexities, key factors including DDR, Rb, p53, and EMT regulators are regularly identified as key actors in the activation of TIS and thus emend the cell cycle process. While current evidence suggests that senescence can be regulated by EMT-associated transcription factors, it remains to be examined whether this connection impacts carcinogenesis and response to therapy.

## Convergence of TIS and EMT

EMT is a process thought to be involved principally in providing spatial and molecular flexibility to tissues during embryonic development and wound healing processes. In the case of cancer, the role of EMT has been related to metastatic dissemination; providing a mechanism for cancer cells to dislodge from their primary site and colonize at distant secondary sites^3^. This process, metastasis, is one of the most feared characteristics of cancer. Senescence, on the other hand, is regarded as a failsafe mechanism to prevent the progression of carcinogenesis and serves as a barrier to attenuate a metastatic phenotype. Senescence and EMT therefore seem to be inherently opposed processes; however, recent evidence has identified key shared signaling points that entwine both processes (Table 2). Further exploration is required to validate senescence and EMT cross-talk as well as identifying their potential as co-druggable targets. In the following section, we will review vital mediators that provide a distinct link between senescence and EMT.

**Table 2:** Common modulators of senescence and EMT.

S. No.	Modulators	Senescence	EMT	References
1	Twist-1	−	+	^38,42,45,46^
2	Twist-2	−	+	^42^
3	Zeb1	−	+	^49,50^
4	Zeb2 (SIP1)	+	+	^51–53^
5	Snail 1	−	+	^54–56^
6	Slug	−	+	^63–66^
7	p21	+	−/+	^22,57,58,64,69,83^
8	p53	+	−	^26–28,30,63,78,81^
9	Rb	+	−	^35,48,50,59,60,62^

‘+’ indicates favouring impetus, ‘−’ indicates opposing impetus.

### EMT-associated transcription factors regulate senescence

The association between major EMT-associated transcriptions factors, including Twist-1, Zeb1, Snail1, and Slug, and the modulation of senescence is of great interest to the scientific community (Fig.^ 2^). Paramount to this association is they study of the targetability of EMT and its ability to predispose cancer cells to TIS. Twist-1, a member of basic helix loop helix (bhlh) family of transcription factors, has been extensively described in developmental processes as well as in progression of metastasis^41^. Twist-1 acts as the repressor of the E-box protein, E-cadherin and members of the *Ink4* locus, p14, p16, and p21 at the promoter level. Notably, Twist-2, also a bhlh member, acts as an *Ink4* locus repressor. In an *Ink4a-ARF*^*-/-*^ mouse embryonic fibroblast (MEF) model, Twist-1 ameliorates N-Myc driven transformation, decreased expression of epithelial markers E-cadherin and claudin, and increases expression of the mesenchymal marker Vimentin^42^. Strikingly, in the same experimental setup, the abolition of senescence-associated markers is distinctly observed with increased Twist-1 activity. Twist-1 reverses p53-dependent cell cycle arrest but also enhances the oncogenic transformation of H-Ras expressing cells. Twist-1 has also been shown to cooperate with mutant K-Ras to facilitate lung tumorigenesis in transgenic mouse models^43^. Silencing of Twist-1 results in K-Ras-mediated senescence^44^ while ectopic Twist-1 forestalls p53 and p21 induction in DNA damage background^42^. The mechanism which would explain the role of Twist-1 in the obstruction of senescence requires further study. In human prostate epithelial cells Twist-1 hinders senescence in p14-dependent manner^39^; however, in gastric cancer cells, Twist-1 knockdown provokes cell cycle arrest induced by p14 in a p53-dependent manner^45^. Of note, Twist-1 has been demonstrated to regulate p53 levels indirectly by repressing p14; however, direct interaction between p53 and Twist-1 and subsequent transactivation of p53 is plausible^46^. γ-irradiation mediated AKT phosphorylation of Twist-1 at ser42 residue leads to cell cycle progression due to cessation of p53 transactivation. Further, in melanoma cells harboring the *BRAF*^*E600*^ mutation, RNAi mediated silencing of Twist-1 promotes activation of senescence^47^. In human epithelial cells and MEFs, exogenous overexpression of oncogenic ERBB2 drives p21 nuclear accumulation and induction of premature senescence; however, Twist-1 has been shown to negate the ERBB2 driven cellular senescence in this setting^48^. Here, oncogenic cooperation between Twist-1 and ERBB2 confers complete EMT activation and functionally bypasses senescence; suggesting the senescence overriding potential of Twist-1^42^. On the basis of this evidence, Twist-1 has been suggested as a prospective therapeutic target. Further effort is necessary to design and identify Twist-1 antagonists that may serve to counter EMT activation and sensitize cancer cells to activation of senescence^40^.

In addition to the mechanisms outlined above, further studies have identified potential alternative connections between other EMT-associated transcription factors and senescence signaling. Zinc finger E-box binding homeobox 1 (Zeb1), a transcription factor that facilitates tumor invasion by augmenting EMT in carcinoma cells, represents a unique obstacle to cancer therapeutics. Zeb1 acts as a negative repressor of E-cadherin. In Zeb1-null MEFs, elevated expression on p21 was found to prevent activation of senescence. The role of Zeb1 in metastatic dissemination has been thoroughly studied; leading to additional findings of its role in subverting cell cycle exit programs: senescence and apoptosis. Partial down-regulation of Zeb1 is sufficient to induce senescence in mouse xenograft models^49^. Mutations in Zeb1 have been demonstrated to drive premature senescence in MEFs via direct induction of p15 and p21 mediated through binding to their respective promoter sites. Additionally, Zeb1 and mir200C expression has been found to be inversely correlated. Yongqing et al. identified a negative feedback loop existing between Zeb1 and mir200C, which co-regulates Bmi 1 expression in cancer cells. Critically, Zeb1 driven induction of Bmi 1 expression is dependent on the Rb status within a given cell. In cells with intact Rb, Zeb1 does not stimulate Bmi 1 expression leading to premature senescence. Alternatively, in cells with altered Rb, Zeb1 driven induction of Bmi 1 leads to activation of EMT^50^. The finding from this study suggests that oncogenes acting in isolation fail to drive carcinogenesis and invariably provoke anti-tumorigenic response or senescence. However, subsequent triggering of other oncogenes, or lack of pivotal tumor suppressors, creates a molecular shift resulting in a pro-tumorigenic environment^9^. Additionally, p16-induced senescence with concomitant EMT inhibition, through mir-141/mir-146b-5p dependent abrogation of Zeb1, demonstrates a lack of a SASP. Notably, a few studies implicate Zeb2 in favoring senescence activation. For example, Zeb2 has been found to be amplified during GADD45G-induced senescence in hepatocellular carcinoma. Additionally, Zeb2 inactivation by RNAi leads to the circumvention of senescence in these cells^51–53^.

Snail1, also a member of the zinc finger family, facilitates EMT and resists entry of cells into senescence. Repression of Snail1 activity induces senescence in addition to diminishing cellular invasion^54^. Snail1 induction, therefore, results in the inhibition of senescence in aggressive human prostate cancer cell lines^55^. However, the dichotomy of Snail1 functionality, with respect to senescence and EMT, cannot be overlooked as the multifaceted role of Snail1 includes strong modulation of p21; effectively resulting in cell cycle arrest^56^. Along with Twist-1, Snail1 inhibits E2A-induced p21 expression to favor EMT activation^57^. Surprisingly, p21 itself possesses a dual role in carcinogenesis. For example, as opposed to the earlier defined tumor-suppressive roles of p21, mounting evidence reveals a non-canonical role of p21 in tumor progression and senescence induction dependent on the cellular localization of p21. In the context of EMT and senescence, RasV12-induced EMT in MCF10A cells results in diminished p21 expression. In vivo studies with transgenic mice expressing RasV12 and deficient in p21 show accelerated development of EMT features^58^. Collectively, these data suggest that initiation of EMT is mostly accompanied by bypass of senescence. Whether this bypass is deliberately induced by EMT-associated transcription factors for the EMT program to begin or just a collateral event during the molecular reprogramming in cancer cells when they undergo EMT remains to be fully comprehended.

### Effectors of senescence regulate EMT

In previous sections we outlined the role of EMT governing transcription factors with senescence. In addition to these mechanisms, there is data that suggests that effectors of senescence also facilitate cross-talk with EMT signaling cascades (Fig.^ 2^). p16-mediated hypophosphorylation of Rb has a well-documented role in sustaining of senescence phenotype^59^. Rb depletion in breast cancer cell lines has been demonstrated to lead to the development of a mesenchymal phenotype through the induction of EMT-related transcription factors Zeb1 and Slug^60^. Furthermore, Rb-mediated E-cadherin repression facilitates EMT in Simian virus 40 infected MDCK epithelial cells^61^. Similarly, bone morphogenetic protein 2 (BMP-2) degrades Rb through ubiquitinylation in breast cancer cell lines as well as clinical samples; thus resulting in the development of breast cancer stem cells (BCSs) and enhancement of EMT signaling^62^. Due to the quintessential role of Rb in senescence, these findings link Rb, senescence, and EMT in a linear axis.

While functional p53 is known to inhibit Slug via MDM2-mediated post-translational degradation, mutant p53 initiates Slug accumulation and increases invasiveness^63^. p53 is often mutated in non-small-cell lung cancer leading to high Slug expression; which then correlates with low MDM2 levels and poor overall survival. Inhibition of Slug transcription by DNA damage sensor protein, hRAD9, drives p21-dependent senescence and suppression of EMT^64^. Interestingly, treatment with 5-FU induces senescence in colon cancer cell line (HCT-116) by enhancing Slug mRNA levels and concomitant stimulation of EMT signaling in a paracrine fashion^65^. Murine p19 and its human counterpart p14 stabilize Slug through sumoylation at the lys19 residue. Stabilized Slug has then been shown to inhibit E-cadherin expression in *PTEN/Trp53* double knockout murine models of prostate cancer^66^. The expression of Slug and p14 are positively correlated in human prostate cancer samples, suggesting an altered senescence pathway can lead to increased tumor progression, particularly in in vivo contexts^66^. The results of the studies presented here suggest numerous potential therapeutic opportunities to promote senescence and mitigate EMT. Further study into the targetability of this axis is warranted.

## Recent trends in therapeutic development targeting TIS

Senescence can be thought of as an endogenous hurdle to malignant transformation. This vital molecular mechanism then provides an opportunity to develop therapeutic strategies to improve therapy options for patients. Numerous studies have identified that premalignant and early cancer cells are more sensitive to pro-senescent drugs than surrounding normal tissue. In premalignant prostatic lesions, intraepithelial neoplastic lesions are frequently identified as senescent. It is therefore reasonable to hypothesize that TIS may serve as a novel approach to cancer management. This is particularly in contexts where apoptotic signaling is disabled or where toxicity is a major hurdle to providing effective therapy.

Genotoxic agents such as doxorubicin, 5-fluorouracil (5-FU), etoposide, camptothecin, cisplatin, and their analogs have been explored for their ability to induce senescence in a wide array of cell types^4^. Notably, the dose of agents necessary to induce TIS is significantly lower than their corresponding cytotoxic dose. In theory, this would also reduce the potential for toxic side-effects associated with these drugs. For example, an in vitro screening of fibrosarcoma cells treated at equi-toxic doses unveiled better senescence provoking potential of DNA-interacting agents, doxorubicin and cisplatin, in comparison to the mitotic catastrophe inducing agent docetaxel^67,68^. In an in vivo breast cancer model, tumors treated with a combination of doxorubicin, cyclophosphamide, and 5-FU reveal distinct populations of SA-β-gal positive cells, a hallmark of senescence activation^67^. Similarly, in lung tumors exposed to combination of carboplatin and docetaxel therapy, molecular markers of senescence are highly elevated following treatments^26^. Recently, we have demonstrated that cristacarpin, a plant-based natural product derived from *Erythrina suberosa*, triggers endoplasmic reticulum stress followed by sub-toxic ROS generation. These result in a p21-mediated G_1_ phase cell cycle arrest; eventually provoking senescence in a p53-independent manner. Additionally, cristacarpin treatment resulted in ROS-dependent activation of the MAP kinase pathway, as noted by increased p38MAPK levels^69^.

Another report from our laboratory identified additional cross-talk between EMT and senescence, wherein the functional role of Chk2 in premature senescence was observed upon treatment with 4′-demethyl-deoxypodophyllotoxin glucoside (4DPG). Interestingly, Chk2-mediated senescence halted EMT signaling in p53-deficient cancer cells in a number of cancer types^40^. Notably, Chk2 induction in p53-mutated invasive cells abrogates tissue invasion, cell scattering, and invadopodia formation ability by suppressing the major EMT regulator Twist-1. This indicates a vital role of Chk2 in senescence induction and metastasis aversion. Treatment with the small molecule inhibitor of Aurora kinase A, MLN 8054, prompts senescence in HCT-116 cells via up-regulation of p53 and p21. Likewise, other inhibitors of Aurora kinase A, AKI603 and MLN8237, trigger senescence in chronic myeloid leukemia cells and metastatic melanoma tumors in murine models; mediated by the ATM/Chk2 axis^70^. Non-steroidal anti-inflammatory drugs, like aspirin (500 µM), induce senescence in colorectal carcinoma cells by targeting SIRT1 and AMPK^71^. AMPK abrogation has been correlated with the nullification of metastatic processes in multiple cancer types^72^. Moreover, STK899204, a novel small molecule, promotes senescence in A549 cells by inducing the DDR pathway leading to cell cycle arrest in the G_2_/M phase^73^.

Recently, small molecule based high-throughput screening for identification of novel TIS agents have been developed based on monitoring of SA-β-gal activity and cellular proliferation^74^. Additionally, two-hit systems like the CRISPR/Cas-9 based genetic screens and high-throughput compound screens developed can serve to identify “synthetic senescence” targets similar to the identification of “synthetic lethality” studies^75^. While several novel senescence-inducing molecules have been unveiled, there is a lack of potential lead molecules that can simultaneously induce senescence while inhibiting EMT. A comprehensive list of agents that induce accelerated TIS and their mechanisms of action is presented in Table 3.

**Table 3:** Modulators of therapy-induced senescence.

S. No.	Molecule	Nature of molecule	Mechanism	Reference
1	Doxorubicin	Cytotoxic anthracycline antibiotic	DNA intercalator induces s by poisoning DNA topoisomerase II	^67,68^
2	Daunorubicin	Anthracycline	DNA intercalator, poisons topoisomerase II	^88^
3	Etoposide	Semisynthetic derivative of podophyllotoxin	Poison of topoisomerase II induces DSBs	^67^
4	Gemcitabine	Pyrimidine nucleoside pro drug	Inhibits ribonucleotidereductase, inhibits CTP synthetase	^89^
5	Camptothecin and SN-38	Alkaloid	TopoisomeraseI poison, induces SSBs	^67^
6	Cisplatin	Platinum based	DNA alkylating agent, induces DNA intra-stand crosslinks	^90^
7	Cyclophosphamide	Cytophosphane	Induces DNA inter and intra-strand crosslinks	^91^
8	Aphidicolin	Tetracyclic diterpene	Inhibitor of DNA polymerase α	^92^
9	Mitoxantrone	Anthracenedione derivative	Topoisomerase II inhibitor	^93^
10	Bromodeoxyuridine	Synthetic nucleoside analog of thymidine	Suppresses DNA replication	^94^
11	Thymidine	Pyrimidine deoxynucleoside	Inhibits DNA replication by reducing amount of dCTP synthesized	^95^
12	Mitomycin c	Mitomycin	DNA alkylating agent induces DNA inter-strand crosslinks	^96^
13	Busulfan	Alkyl sulfonate	Induces DNA intra-strand crosslinks	^97^
14	Hydroxyurea	Hydroxycarbamide	Ribonucleotidereductase inhibitor	^92^
15	Diaziquone	Synthetic aziridinylbenzoquinone	Induces DNA-DNA and DNA -RNA inter-strand cross links	^98^
16	Actinomycin d	Cyclic peptide	DNA inter-calator, inhibits transcription	^99^
17	Bleomycin	Peptide	Induces DNA breaks	^100^
18	Temozolomide	Alkylating agent	Alkylates/methylates DNA, induces DNA damage	^101^
19	5-aza-2′-deoxycytidine	Cytidine analog	Inhibitor of DNAmethyltransferases,Induces DSBs	^102^
20	Sodium butyrate	Sodium salt of butyric acid	Class I and II HDAC inhibitor	^103^
21	Trichostatin a	Dienohydroxamic acid derivative	Class I and II HDAC inhibitor	^17^
22	Ms-275	Benzamide derivative	Class I HDAC inhibitor	^104^
23	Saha (Vorinostat)	Suberanilohydroxamic acid	Class I and II HDAC inhibitor	^104^
24	Lbh589 (Panobinostat)	Hydroxamic acid	Class I and II HDAC inhibitor	^105^
25	4-phenylbutyric acid	Mono-carboxylic acid	Class I and IIa HDAC inhibitor	^106^
26	Valproic acid	Fatty acid	Class I and IIa HDAC inhibitor	^107^
27	Curcumin and c646	Curcuminoid	P300 Histone acetyltransferase Inhibitor	^108^
28	Brd4770	Carboxylic acid	Histone methyltransferase inhibitor	^109^
29	Syuiq-5	Cryptolepina derivative	Stabilizes g-quadruplexes, induces Trf2 delocalization from telomeres	^110^
30	Bmvc4	Carbazole derivative	Stabilizes g-quadruplexes	^111^
31	Pyridostatin	Trifluoroacetate salt	Stabilizes g-quadruplexes	^112^
32	Compound 115405	Peptide	G-quadruplex ligand	^113^
33	Pm2 andPiper	Perylene derivative	Induces g-quadruplex formation from both telomeric DNA and htert promoter region	^114^
34	Harmine	Alkaloid	β-carboline alkaloid	^115^
35	Bibr1532	Synthetic non-nucleosidic derivative	Non-nucleosidictert inhibitor	^116^
36	Azidothymidine (AZT)	Dideoxynucleoside	Reverse transcriptase inhibitor, inhibits telomerase activity	^117^
37	Palbociclib (PD-0332991)	Pyridopyrimidine	Cdk4 and Cdk6 inhibitor	^118^
38	Roscovitine (seliciclib)	Purine analog	Cdk2, Cdk7, and Cdk9 inhibitor	^119^
39	Ribociclib (LEE011)	Tartrate salt	Cdk4 and Cdk6 inhibitors	^120^
40	Nutlin-3a	Cis-imidazoline analog	Inhibits MDM2 binding to p53	^121^
41	Fl118	Camptothecin derivative	Proteasomal degradation of MDMX	^122^
42	Pep005 (ingenol-3-angelate)	Ester of diterpeneingenol and angelic acid	Activates PKC	^123^
43	MLN8054, MLN8237	Small molecule	Aurora kinase A inhibitors	^70^

## Limitations

In cancer, the biologic cycle of senescence/clearance/regeneration is highly altered. Senescence and SASP are thus posed to be detrimental due to the associated auto and paracrine effects. Furthermore, this leads to loss of tissue functionality and remodeling, chronic inflammation, and advancement of SASP; thus fueling a pro-carcinogenic microenvironment. Cellular senescence can trigger tumorigenesis by enhancing SASP and modulating the extracellular milieu; resulting in heightened proliferation and invasion potential^11^. Cells undergoing apoptosis in response to cytotoxic drugs are readily eliminated; however, senescent cells can persist indefinitely despite exposure to cytotoxic drugs. For example, in papillary thyroid carcinoma (PTC), senescent tumor cells enhance the invasive potential by switching on SASP through CXCL12/ CXCR4C^76^. However, senescence as a pro-tumorigenic process is entirely context-dependent and there is a broad consensus that TIS primarily has tumor-repressive roles. For instance, isolated senescent cells are indeed present in invasive cancers, whereas pre-malignant tumors rarely show any sign of senescent cells^76^. Similarly, senescence-associated cytokines, IL-6 and IL-8, can reinforce senescence in MCF7 cells leading to a pro-inflammatory and tumorigenic milieu^77^. Further, conditional media from senescent cells significantly reduces the cell surface expression of β-catenin and E-cadherin complexes as well as increased nuclear localization of claudin; thus rendering the cells prone to EMT development^11,78^. Despite the dearth of evidence suggesting the therapeutic potential of TIS and EMT, further research is necessary to improve our understanding of this complex biologic pathway.

## Future directions

The primary goal of clinical cancer management is to provide the highest quality of life for the longest amount of time possible for a given patient. Traditional therapeutic methods in cancer are focused on total obliteration of cancer cells which rarely achieves this goal. The heterogeneity of the cells in terminally differentiated cancers poses a significant challenge to traditional chemotherapeutic agents; often leading to the formation or selection of resistant sub-clones. Compelling evidences also suggest that cytotoxic drugs may render a subset of cancer cells with an invasive and migratory phenotype. Therapeutic exploitation of senescence-associated vulnerabilities can help to achieve a state in cancer management where further aggravation of disease burden is checked while limiting the proliferative capacity of cancer cells. This paradigm shift in treating cancer as a chronic disease may render a favorable outcome for patients in the long term compared with traditional therapeutic techniques.

Senescence then serves as a promising alternative therapeutic goal in cancer management as stabilizing tumor burdens may provide improved overall survival. TIS not only creates a state of cytostasis in cancer cells but renders them susceptible to cytotoxic drugs at far lower doses than normal. Interestingly, cancer cells that have altered tumor-suppressive pathways remain sensitive to TIS, suggesting its broad applicability. Apart from cytostasis, other advantages of TIS include immunogenic stimulation and relatively low toxicity. Additionally, with the advent of ‘senolytics’, drugs that specifically clear senescent cells, rational combinatorial therapy can be designed that overcome the deleterious effects associated with long-term SASP production.

It is clear then, based on the data available, that cross-talk between senescence and EMT signaling are key to each biological function. In activating either of these pathways, the other seems to be biologically required to be inactivated. The causation or correlations between these two pathways have not yet been fully demonstrated. Acquisition of EMT may not always be accompanied by the bypass of senescence; however the literature suggests that these processes are intrinsically linked. With the identification of novel signaling intersections between these programs the opportunity to intervene therapeutically presents a promising field of study. Regardless, identification of senescence and EMT as mutually inclusive programs is still ambiguous. Differential regulation of EMT-associated transcription factors or the effectors of senescence is context dependent and yet to be fully understood. Identification of novel senescence-inducing agents that can potentially hamper EMT activation is also yet to be realized. There is significant need to address these issues, which will eventually aid in devising improved therapeutic strategies for cancer management.

## References

[1] Housman G (2014). Drug resistance in cancer: an overview. Cancers.

[2] Holohan C, Van Schaeybroeck S, Longley DB, Johnston PG (2013). Cancer drug resistance: an evolving paradigm. Nat. Rev. Cancer.

[3] Kalluri R, Weinberg RA (2010). The basics of epithelial-mesenchymal transition. J. Clin. Investig..

[4] Ewald JA, Desotelle JA, Wilding G, Jarrard DF (2010). Therapy-induced senescence in cancer. J. Natl Cancer Inst..

[5] Schmitt CA (1775). Cellular senescence and cancer treatment. BBA-Rev. Cancer.

[6] Collado M, Blasco MA, Serrano M (2007). Cellular senescence in cancer and aging. Cell.

[7] Shay JW, Wright WE (2000). Hayflick, his limit, and cellular ageing. Nat. Rev. Mol. Cell Biol..

[8] Terry A, Cameron E, Neil J, Kilbey A (2008). Oncogene-induced senescence: an essential role for Runx. Cell Cycle.

[9] Kuilman T, Michaloglou C, Mooi WJ, Peeper DS (2010). The essence of senescence. Genes Dev..

[10] Schönlein M (2017). Therapy induced senescence is a predictor of treatment outcome in acute myeloid leukemia. Blood.

[11] Coppé J-P, Desprez P-Y, Krtolica A, Campisi J (2010). The senescence-associated secretory phenotype: the dark side of tumor suppression. Annu. Rev. Pathol. -Mech. Dis..

[12] Lopes-Paciencia S (2019). The senescence-associated secretory phenotype and its regulation. Cytokine.

[13] Rodier F, Campisi J (2011). Four faces of cellular senescence. J. Cell Biol..

[14] Lecot P, Alimirah F, Desprez P-Y, Campisi J, Wiley C (2016). Context-dependent effects of cellular senescence in cancer development. Br. J. Cancer.

[15] Brabletz T, Kalluri R, Nieto MA, Weinberg RA (2018). EMT in cancer. Nat. Rev. Cancer.

[16] Mallette FA, Gaumont-Leclerc M-F, Ferbeyre G (2007). The DNA damage signaling pathway is a critical mediator of oncogene-induced senescence. Genes Dev..

[17] Munro J, Barr NI, Ireland H, Morrison V, Parkinson EK (2004). Histone deacetylase inhibitors induce a senescence-like state in human cells by a p16-dependent mechanism that is independent of a mitotic clock. Exp. Cell. Res..

[18] Miller KM (2010). Human HDAC1 and HDAC2 function in the DNA-damage response to promote DNA nonhomologous end-joining. Nat. Struct. Mol. Biol..

[19] Jackson SP, Bartek J (2009). The DNA-damage response in human biology and disease. Nature.

[20] Zhou B-BS, Elledge SJ (2000). The DNA damage response: putting checkpoints in perspective. Nature.

[21] Schwartz GK, Shah MA (2005). Targeting the cell cycle: a new approach to cancer therapy. J. Clin. Oncol..

[22] Campisi J (2013). Aging, cellular senescence, and cancer. Annu. Rev. Physiol..

[23] Litwiniec A, Gackowska L, Helmin-Basa A, Żuryń A, Grzanka A (2013). Low-dose etoposide-treatment induces endoreplication and cell death accompanied by cytoskeletal alterations in A549 cells: does the response involve senescence? The possible role of vimentin. Cancer Cell Int.

[24] Sharpless NE (2004). Ink4a/Arf links senescence and aging. Exp. Gerontol..

[25] Narita M (2003). Rb-mediated heterochromatin formation and silencing of E2F target genes during cellular senescence. Cell.

[26] Roberson RS, Kussick SJ, Vallieres E, Chen S-YJ, Wu DY (2005). Escape from therapy-induced accelerated cellular senescence in p53-null lung cancer cells and in human lung cancers. Cancer Res..

[27] Serrano M, Lin AW, McCurrach ME, Beach D, Lowe SW (1997). Oncogenic ras provokes premature cell senescence associated with accumulation of p53 and p16INK4a. Cell.

[28] Calabrese V (2009). SOCS1 links cytokine signaling to p53 and senescence. Mol. Cell.

[29] Brooks CL, Gu W (2011). p53 regulation by ubiquitin. FEBS Lett..

[30] Rufini A, Tucci P, Celardo I, Melino G (2013). Senescence and aging: the critical roles of p53. Oncogene.

[31] Chicas A (2010). Dissecting the unique role of the retinoblastoma tumor suppressor during cellular senescence. Cancer Cell.

[32] Rayess H, Wang MB, Srivatsan ES (2012). Cellular senescence and tumor suppressor gene p16. Int. J. Cancer.

[33] Hitomi T (2007). Oct‐1 is involved in the transcriptional repression of the p15INK4b gene. FEBS Lett..

[34] Robles SJ, Adami GR (1998). Agents that cause DNA double strand breaks lead to p16 INK4a enrichment and the premature senescence of normal fibroblasts. Oncogene.

[35] Zhang Y, Xiong Y, Yarbrough WG (1998). ARF promotes MDM2 degradation and stabilizes p53: ARF-INK4a locus deletion impairs both the Rb and p53 tumor suppression pathways. Cell.

[36] Wiley CD (2018). Small-molecule MDM2 antagonists attenuate the senescence-associated secretory phenotype. Sci. Rep..

[37] Seligson ND (2019). Inhibition of histone deacetylase 2 reduces MDM2 expression and reduces tumor growth in dedifferentiated liposarcoma. Oncotarget.

[38] Puisieux A, Valsesia-Wittmann S, Ansieau S (2006). A twist for survival and cancer progression. Br. J. Cancer.

[39] Kwok WK, Ling M-T, Yuen HF, Wong Y-C, Wang X (2007). Role of p14 ARF in TWIST-mediated senescence in prostate epithelial cells. Carcinogenesis.

[40] Nayak D (2017). Inhibition of Twist1-mediated invasion by Chk2 promotes premature senescence in p53-defective cancer cells. Cell Death Differ..

[41] Yang J (2004). Twist, a master regulator of morphogenesis, plays an essential role in tumor metastasis. Cell.

[42] Ansieau S (2008). Induction of EMT by twist proteins as a collateral effect of tumor-promoting inactivation of premature senescence. Cancer Cell.

[43] Burns TF (2013). Inhibition of TWIST1 leads to activation of oncogene-induced senescence in oncogene-driven non–small cell lung cancer. Mol. Cancer Res..

[44] Tran PT (2012). Twist1 suppresses senescence programs and thereby accelerates and maintains mutant Kras-induced lung tumorigenesis. PLoS Genet..

[45] Feng M-y (2009). Metastasis-induction and apoptosis-protection by TWIST in gastric cancer cells. Clin. Exp. Metastasis.

[46] Shiota M (2008). Twist and p53 reciprocally regulate target genes via direct interaction. Oncogene.

[47] Michaloglou C (2005). BRAF E600-associated senescence-like cell cycle arrest of human naevi. Nature.

[48] Trost TM (2005). Premature senescence is a primary fail-safe mechanism of ERBB2-driven tumorigenesis in breast carcinoma cells. Cancer Res..

[49] De Barrios O (2017). ZEB1-induced tumourigenesis requires senescence inhibition via activation of DKK1/mutant p53/Mdm2/CtBP and repression of macroH2A1. Gut.

[50] Liu Y (2013). Sequential inductions of the ZEB1 transcription factor caused by mutation of Rb and then Ras proteins are required for tumor initiation and progression. J. Biol. Chem..

[51] Xu G (2015). SIP1 is a downstream effector of GADD45G in senescence induction and growth inhibition of liver tumor cells. Oncotarget.

[52] Ozturk N (2006). Reprogramming of replicative senescence in hepatocellular carcinoma-derived cells. Proc. Natl Acad. Sci. USA.

[53] Al-Khalaf HH (2011). p16INK4a positively regulates cyclin D1 and E2F1 through negative control of AUF1. PLoS ONE.

[54] Furuya S, Endo K, Takahashi A, Miyazawa K, Saitoh M (2017). Snail suppresses cellular senescence and promotes fibroblast‐led cancer cell invasion. FEBS Open Bio.

[55] Baygi ME, Soheili ZS, Schmitz I, Sameie S, Schulz WA (2010). Snail regulates cell survival and inhibits cellular senescence in human metastatic prostate cancer cell lines. Cell Biol. Toxicol..

[56] Vega S (2004). Snail blocks the cell cycle and confers resistance to cell death. Genes Dev..

[57] Takahashi E (2004). Snail regulates p21WAF/CIP1 expression in cooperation with E2A and Twist. Biochem. Biophys. Res. Commun..

[58] Navaraj A (2009). Reduced cell death, invasive and angiogenic features conferred by BRCA1-deficiency in mammary epithelial cells transformed with H-Ras. Cancer Biol. Ther..

[59] Takahashi A, Ohtani N, Hara E (2007). Irreversibility of cellular senescence: dual roles of p16 INK4a/Rb-pathway in cell cycle control. Cell Div..

[60] Arima Y (2012). Induction of ZEB proteins by inactivation of RB protein is key determinant of mesenchymal phenotype of breast cancer. J. Biol. Chem..

[61] Larsson O (2004). Kinetics of senescence-associated changes of gene expression in an epithelial, temperature-sensitive SV40 large T antigen model. Cancer Res..

[62] Huang P (2017). BMP-2 induces EMT and breast cancer stemness through Rb and CD44. Cell Death Discov..

[63] Wang S-P (2009). p53 controls cancer cell invasion by inducing the MDM2-mediated degradation of Slug. Nat. Cell Biol..

[64] Wen F-C, Chang T-W, Tseng Y-L, Lee J-C, Chang M-C (2014). hRAD9 functions as a tumor suppressor by inducing p21-dependent senescence and suppressing epithelial–mesenchymal transition through inhibition of Slug transcription. Carcinogenesis.

[65] Tato-Costa J (2016). Therapy-induced cellular senescence induces epithelial-to-mesenchymal transition and increases invasiveness in rectal cancer. Clin. Colorectal Cancer.

[66] Xie Y (2014). Slug regulates E‐cadherin repression via p19Arf in prostate tumorigenesis. Mol. Oncol..

[67] te Poele RH, Okorokov AL, Jardine L, Cummings J, Joel SP (2002). DNA damage is able to induce senescence in tumor cells in vitro and in vivo. Cancer Res..

[68] Chang B-D (1999). A senescence-like phenotype distinguishes tumor cells that undergo terminal proliferation arrest after exposure to anticancer agents. Cancer Res..

[69] Chakraborty S (2016). Cristacarpin promotes ER stress-mediated ROS generation leading to premature senescence by activation of p21 waf-1. Age.

[70] Huck JJ (2010). MLN8054, an inhibitor of Aurora A kinase, induces senescence in human tumor cells both in vitro and in vivo. Mol. Cancer Res..

[71] Jung YR (2015). Aspirin targets SIRT1 and AMPK to induce senescence of colorectal carcinoma cells. Mol. Pharmacol..

[72] Seah KS (2018). SAHA and cisplatin sensitize gastric cancer cells to doxorubicin by induction of DNA damage, apoptosis and perturbation of AMPK-mTOR signalling. Exp. Cell Res..

[73] Park C-W (2018). The novel small molecule STK899704 promotes senescence of the human A549 NSCLC cells by inducing DNA damage responses and cell cycle arrest. Front. Pharmacol..

[74] Bitler BG, Fink LS, Wei Z, Peterson JR, Zhang R (2013). A high-content screening assay for small-molecule modulators of oncogene-induced senescence. J. Biomol. Screen..

[75] Wang L (2017). High-throughput functional genetic and compound screens identify targets for senescence induction in cancer. Cell Rep..

[76] Kim YH (2017). Senescent tumor cells lead the collective invasion in thyroid cancer. Nat. Commun..

[77] Ortiz-Montero P, Londoño-Vallejo A, Vernot J-P (2017). Senescence-associated IL-6 and IL-8 cytokines induce a self-and cross-reinforced senescence/inflammatory milieu strengthening tumorigenic capabilities in the MCF-7 breast cancer cell line. Cell Commun. Signal..

[78] Coppé J-P (2008). Senescence-associated secretory phenotypes reveal cell-nonautonomous functions of oncogenic RAS and the p53 tumor suppressor. PLoS Biol..

[79] Wei W, Hemmer RM, Sedivy JM (2001). Role of p14ARF in replicative and induced senescence of human fibroblasts. Mol. Cell. Biol..

[80] Erickson S (1998). Involvement of the Ink4 proteins p16 and p15 in T-lymphocyte senescence. Oncogene.

[81] Beauséjour CM (2003). Reversal of human cellular senescence: roles of the p53 and p16 pathways. EMBO J..

[82] Gagrica S, Brookes S, Anderton E, Rowe J, Peters G (2012). Contrasting behavior of the p18INK4c and p16INK4a tumor suppressors in both replicative and oncogene-induced senescence. Cancer Res..

[83] Abbas T, Dutta A (2009). p21 in cancer: intricate networks and multiple activities. Nat. Rev. Cancer.

[84] Alexander K, Hinds PW (2001). Requirement for p27KIP1 in retinoblastoma protein-mediated senescence. Mol. Cell. Biol..

[85] Tang H (2015). NSun2 delays replicative senescence by repressing p27 (KIP1) translation and elevating CDK1 translation. Aging.

[86] Giovannini C (2012). CDKN1C/P57 is regulated by the Notch target gene Hes1 and induces senescence in human hepatocellular carcinoma. Am. J. Pathol..

[87] Tsugu A (2000). Expression of p57KIP2 potently blocks the growth of human astrocytomas and induces cell senescence. Am. J. Pathol..

[88] Mansilla S, Benjamin P, Portugal J (2003). Daunorubicin-induced variations in gene transcription: commitment to proliferation arrest, senescence and apoptosis. Biochem. J..

[89] Modrak DE, Leon E, Goldenberg DM, Gold DV (2009). Ceramide regulates gemcitabine-induced senescence and apoptosis in human pancreatic cancer cell lines. Mol. Cancer Res..

[90] Wei Z, Lin ZX, Zhang ZQ (2004). Cisplatin-induced premature senescence with concomitant reduction of gap junctions in human fibroblasts. Cell Res..

[91] Palaniyappan A (2009). Cyclophosphamide induces premature senescence in normal human fibroblasts by activating MAP kinases. Biogerontology.

[92] Marusyk A, Wheeler LJ, Mathews CK, DeGregori J (2007). p53 mediates senescence-like arrest induced by chronic replicational stress. Mol. Cell. Biol..

[93] Seifrtova M (2013). Mitoxantrone ability to induce premature senescence in human dental pulp stem cells and human dermal fibroblasts. J. Physiol. Pharmacol..

[94] Suzuki T (2001). Induction of senescence-associated genes by 5-bromodeoxyuridine in HeLa cells. Exp. Gerontol..

[95] Sumikawa E, Matsumoto Y, Sakemura R, Fujii M, Ayusawa D (2005). Prolonged unbalanced growth induces cellular senescence markers linked with mechano transduction in normal and tumor cells. Biochem. Biophys. Res. Commun..

[96] McKenna E, Traganos F, Zhao H, Darzynkiewicz Z (2012). Persistent DNA damage caused by low levels of mitomycin C induces irreversible cell senescence. Cell Cycle.

[97] Probin V, Wang Y, Zhou D (2007). Busulfan-induced senescence is dependent on ROS production upstream of the MAPK pathway. Free Radic. Biol. Med..

[98] Ewald JA, Jarrard DF (2012). Decreased skp2 expression is necessary but not sufficient for therapy-induced senescence in prostate cancer. Transl. Oncol..

[99] Minieri V (2015). Persistent DNA damage‐induced premature senescence alters the functional features of human bone marrow mesenchymal stem cells. J. Cell. Mol. Med..

[100] Aoshiba K, Tsuji T, Nagai A (2003). Bleomycin induces cellular senescence in alveolar epithelial cells. Eur. Respir. J..

[101] Aasland D (2019). Temozolomide induces senescence and repression of DNA repair pathways in glioblastoma cells via activation of ATR–CHK1, p21, and NF-κB. Cancer Res..

[102] Venturelli S (2013). Differential induction of apoptosis and senescence by the DNA methyltransferase inhibitors 5-azacytidine and 5-aza-2′-deoxycytidine in solid tumor cells. Mol. Cancer Ther..

[103] Nakagawa H, Sasagawa S, Itoh K (2018). Sodium butyrate induces senescence and inhibits the invasiveness of glioblastoma cells. Oncol. Lett..

[104] Di Bernardo G (2009). Histone deacetylase inhibitors promote apoptosis and senescence in human mesenchymal stem cells. Stem Cells Dev..

[105] Watkins, D. N. Sustained low-dose treatment with the histone deacetylase inhibitor LBH589 induces terminal differentiation of osteosarcoma cells. *Sarcoma* 2013 (2013). 10.1155/2013/608964 PMC3603321 23533324

[106] Kim HD, Jang C-Y, Choe JM, Sohn J, Kim J (2012). Phenylbutyric acid induces the cellular senescence through an Akt/p21WAF1 signaling pathway. Biochem. Biophys. Res. Commun..

[107] Li X-N (2005). Valproic acid induces growth arrest, apoptosis, and senescence in medulloblastomas by increasing histone hyperacetylation and regulating expression of p21Cip1, CDK4, and CMYC. Mol. Cancer Ther..

[108] Grabowska W (2015). Curcumin induces senescence of primary human cells building the vasculature in a DNA damage and ATM-independent manner. Age.

[109] Yuan Y (2012). A small-molecule probe of the histone methyltransferase G9a induces cellular senescence in pancreatic adenocarcinoma. ACS Chem. Biol..

[110] Zhou J (2006). Senescence and telomere shortening induced by novel potent G-quadruplex interactive agents, quindoline derivatives, in human cancer cell lines. Oncogene.

[111] Huang FC, Chang CC, Wang JM, Chang TC, Lin JJ (2012). Induction of senescence in cancer cells by the G‐quadruplex stabilizer, BMVC4, is independent of its telomerase inhibitory activity. Br. J. Phamrmacol.

[112] Müller S (2012). Pyridostatin analogues promote telomere dysfunction and long-term growth inhibition in human cancer cells. Org. Biomol. Chem..

[113] Riou J (2002). Cell senescence and telomere shortening induced by a new series of specific G-quadruplex DNA ligands. Proc. Natl Acad. Sci. USA.

[114] Taka T (2013). Telomere shortening and cell senescence induced by perylene derivatives in A549 human lung cancer cells. Bioorg. Med. Chem..

[115] Zhao L, Wink M (2013). The β-carboline alkaloid harmine inhibits telomerase activity of MCF-7 cells by down-regulating hTERT mRNA expression accompanied by an accelerated senescent phenotype. PeerJ.

[116] Pascolo E (2002). Mechanism of human telomerase inhibition by BIBR1532, a synthetic, non-nucleosidic drug candidate. J. Biol. Chem..

[117] Li H (2006). Effect of 3-azido-3-deoxythymidine (AZT) on telomerase activity and proliferation of HO-8910 cell line of ovarian cancer. Int. J. Biomed. Sci..

[118] Bollard J (2017). Palbociclib (PD-0332991), a selective CDK4/6 inhibitor, restricts tumour growth in preclinical models of hepatocellular carcinoma. Gut.

[119] Crescenzi E, Palumbo G, Brady HJ (2005). Roscovitine modulates DNA repair and senescence: implications for combination chemotherapy. Clin. Cancer Res..

[120] Tripathy D, Bardia A, Sellers WR (2017). Ribociclib (LEE011): mechanism of action and clinical impact of this selective cyclin-dependent kinase 4/6 inhibitor in various solid tumors. Clin. Cancer Res..

[121] Villalonga-Planells R (2011). Activation of p53 by nutlin-3a induces apoptosis and cellular senescence in human glioblastoma multiforme. PLoS ONE.

[122] Ling X (2014). FL118 induces p53-dependent senescence in colorectal cancer cells by promoting degradation of MdmX. Cancer Res..

[123] Ersvaer E (2010). The protein kinase C agonist PEP005 (ingenol 3-angelate) in the treatment of human cancer: a balance between efficacy and toxicity. Toxins.

